# Microbiota in the Tumor Metastatic Cascade: Emerging Evidence Across Invasion, Circulation, and Colonization

**DOI:** 10.3390/ijms27146308

**Published:** 2026-07-15

**Authors:** Yuhang Fang, Shuhan Yang, Yi Xie, Yan Wang, Bailu Sui, Yu Chen, Xinhe Yuan, Ying Zhang

**Affiliations:** 1 Department of Oncology, Guang’anmen Hospital, China Academy of Chinese Medical Sciences, Beijing 100053, China; 20220931904@bucm.edu.cn (Y.F.); xiey199809@163.com (Y.X.); wangyan1wyw@163.com (Y.W.); 2 Graduate School, Beijing University of Chinese Medicine, Beijing 100029, China; 18810055172@163.com (S.Y.); sbl011169@163.com (B.S.); cy18810536825@163.com (Y.C.); bucmyuanxinhe@163.com (X.Y.)

**Keywords:** microbiota, metastatic cascade, invasion, colonization, pre-metastatic niche

## Abstract

Emerging evidence suggests that the microbiota may influence cancer metastasis and has been associated with multiple stages of the metastatic cascade. This review synthesizes recent advances using a unified framework centered on the three main stages of metastasis: invasion, circulation, and colonization. We highlight evidence linking several microbial taxa, including *Fusobacterium nucleatum*, enterotoxigenic *Bacteroides fragilis*, and *Escherichia coli*, to metastatic progression, and discuss how they may promote metastasis through distinct mechanisms in specific contexts. We further summarize stage-specific evidence indicating that the microbiota may contribute to local invasion, hematogenous dissemination, and distant colonization, offering a perspective on microbiota-associated metastatic progression. Finally, we discuss major limitations of the current literature, including the preponderance of preclinical and correlative evidence, constraints on causal inference, cancer-type and model dependence, low-biomass contamination, and open questions in interpreting tumor- and blood-derived microbial signals.

## 1. Introduction

Malignant tumors represent a major global public health issue. According to the latest estimates released by the International Agency for Research on Cancer, the global number of newly diagnosed cancer cases in 2022 was nearly 20 million, with approximately 9.7 million cancer-related deaths [1]. The clinical efficacy of new therapies has improved significantly in recent years, but the five-year survival rate for patients with distant metastasis remains below 40% [2]. Metastasis is the primary cause of treatment failure and mortality in most cancer patients [3]. Tumor metastasis is a multistep process known as the metastatic cascade [4,5]. As a core mechanism underlying tumor progression, metastasis can be broadly divided into three major stages: invasion, circulation, and colonization. In this process, cancer cells break through the basement membrane from the primary tumor site, disseminate through blood vessels and lymphatic vessels or invade directly into local tissues, and finally colonize distant organs to form new metastatic lesions [6]. This raises an important question: whether biological factors beyond tumor cell-intrinsic programs may influence different steps of the metastatic cascade.

The presence of microbiota in human tumors was first reported a century ago, and such microorganisms have been detected in multiple solid tumor types [7]. Indeed, tumor tissues exhibit enrichment of specific microbes and significant differences in microbial composition compared with adjacent normal tissues [8,9,10,11]. These observations, such as those between *Helicobacter pylori* and gastric cancer (GC), or between *Salmonella* and gallbladder cancer, support a close link between the microbiota and tumorigenesis [12,13]. Additional studies have indicated that the microbiota is involved in the metastasis of multiple malignancies, including colorectal, breast, and ovarian cancer [14,15,16,17], through mechanisms involving inflammation, immunosuppression, and DNA damage [18,19,20,21,22,23]. A prototypical example is *Fusobacterium nucleatum* (*F. nucleatum*), which is associated with larger tumor size, advanced tumor stage, and poor prognosis [24,25,26,27]. This bacterium has been shown to promote tumor progression in experimental models of multiple cancers, including colorectal cancer (CRC), breast cancer (BC), oral cancer, GC, and esophageal cancer [28,29,30,31,32].

Collectively, these findings suggest that the microbiota may influence metastatic progression in specific biological contexts. However, current evidence remains largely fragmented, and a systematic understanding of how the microbiota may modulate the sequential stages of the metastatic cascade is still lacking, particularly given the heterogeneity and methodological challenges in this field. Therefore, this review adopts the metastatic cascade as a framework to systematically examine the potential roles and proposed mechanisms of the microbiota across the three stages of metastasis, namely invasion, circulation, and colonization. This review aims to provide an integrated understanding of microbiota-associated metastatic progression, highlight the microbiota as a potential context-dependent modulator across multiple stages of the metastatic cascade, and summarize key limitations of current research.

## 2. Potential Roles of the Microbiota in Local Invasion and Metastatic Initiation

**Figure 1:**
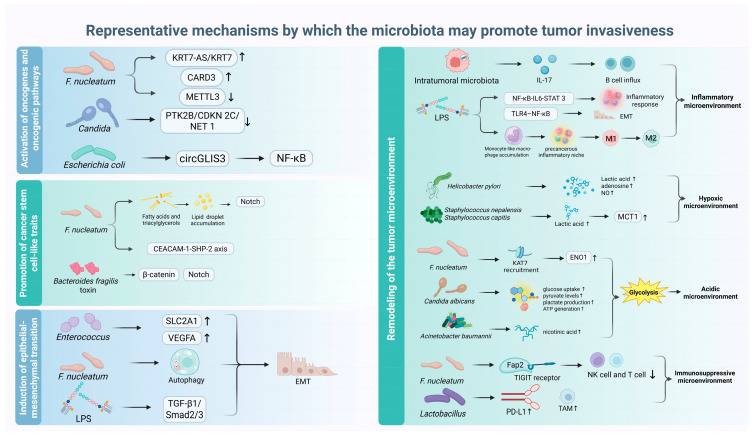
Representative mechanisms by which the microbiota may promote tumor invasiveness. The microbiota may contribute to local invasion and metastatic initiation by remodeling the intrinsic characteristics of cancer cells and the extrinsic microenvironment. Proposed mechanisms include: (1) activation of oncogenes and oncogenic pathways; (2) promotion of cancer stem cell-like traits; (3) induction of epithelial–mesenchymal transition; and (4) remodeling of the inflammatory, hypoxic, acidic, and immunosuppressive tumor microenvironment. Together, these processes may enhance cancer cell invasiveness. The microbial species presented are representative rather than exhaustive, and distinct microbial taxa or components may converge on common pro-metastatic pathways. Created in BioRender. Yuhang, F. (2026) https://BioRender.com/hjoczr6 (accessed on 27 March 2026).

The microbiota may contribute to local invasion and metastatic initiation by remodeling both the intrinsic properties of cancer cells and the extrinsic microenvironment [33]. Microbial colonization within tumors may increase the invasive potential of tumor cells, potentially through the following four mechanisms (Figure 1).

### 2.1. Activation of Oncogenes and Oncogenic Pathways

Emerging studies suggest that the microbiota may contribute to metastatic progression by modulating oncogenes and oncogenic pathways in vitro and in vivo. In CRC, *F. nucleatum* has been reported to promote metastatic progression through mechanisms including upregulating KRT7-AS/KRT7 expression, activating autophagic signaling via CARD3 upregulation, and reducing METTL3 transcription [34,35,36]. In colon cancer, *Candida* has been reported to correlate with downregulated genes involved in cell adhesion and tumor suppression (e.g., PTK2B, CDKN2C, and NET1), potentially enhancing metastatic tendency [37]. In intrahepatic cholangiocarcinoma, *Escherichia coli* induces the expression of the novel circular RNA circGLIS3, which promotes stress granule assembly and activates the nuclear factor kappa-B (NF-κB) signaling pathway, thereby potentially promoting tumor metastasis [38]. Furthermore, the bacterial metabolite butyrate upregulates the long non-coding RNA H19 to facilitate lung cancer (LC) metastasis [39].

### 2.2. Promotion of Cancer Stem Cell (CSC)-like Traits

CSCs exhibit self-renewal and tumorigenic capabilities and are considered important for tumor metastasis [40]. Preclinical evidence links the microbiota to the acquisition of CSC-like traits. *F. nucleatum* has been reported to promote the synthesis of fatty acids and triacylglycerols in differentiated tumor cells, thereby driving lipid droplet accumulation [41]; lipid droplets have been associated with CSC features [42]. This lipid droplet accumulation further activates the Notch signaling pathway to promote the gain of stem-like cell features [41]. Moreover, *F. nucleatum* can enhance fatty acid oxidation in CSCs, which may promote adenosine triphosphate (ATP) production to fuel self-renewal [41,43]. Additionally, in vitro studies have shown that *F. nucleatum* binds to CSCs, triggering pro-inflammatory and oncogenic responses via the CEACAM-1-SHP-2 axis [44]. Similarly, *Bacteroides fragilis* toxin has been shown to elicit morphological and functional alterations in normal mammary epithelial cells and BC cells through the β-catenin and Notch pathways, conferring stem cell-like traits and thereby potentially contributing to metastatic progression [45].

### 2.3. Induction of Epithelial–Mesenchymal Transition (EMT)

EMT is a cellular process that endows cancer cells with mesenchymal phenotypes and enhanced migratory capacity, reduces cell-cell adhesion, and thereby increases their invasive and metastatic potential [46]. Accumulating evidence, predominantly from preclinical studies with support from some clinical observations, suggests that the microbiota may induce EMT and thereby facilitate the dissemination of cancer cells from the primary site. For instance, in hepatocellular carcinoma, *Enterococcus* abundance is correlated with EMT-related genes such as SLC2A1 and VEGFA, suggesting it may promote tumor cell migration and invasion by regulating the EMT process [47]. In oral squamous cell carcinoma, outer membrane vesicles secreted by *F. nucleatum* regulate the expression of EMT-related proteins by activating autophagic pathways, thereby facilitating the invasion and migration of tumor cells [48]. Gram-negative bacteria are a source of lipopolysaccharide (LPS) [49]. LPS triggers EMT in normal human intrahepatic biliary epithelial cells through activation of the transforming growth factor-β1/Smad2/3 signaling pathway [50].

### 2.4. Remodeling of the Tumor Microenvironment (TME)

The TME exhibits characteristic features such as inflammation, hypoxia, acidity, and immunosuppression [51,52]. Accumulating evidence, largely from preclinical studies with support from some clinical observations, suggests that the microbiota may help shape a microenvironment that favors tumor growth, invasion, and metastasis.

#### 2.4.1. Promotion of an Inflammatory Microenvironment

Inflammation is a key factor associated with tumor metastasis [53,54,55], and the microbiota is a significant trigger of tumor-related inflammation. The microbiota can orchestrate a pro-tumor inflammatory microenvironment that supports metastatic progression. For instance, in a mouse model of colitis-associated colon cancer, intratumoral microbiota in neutrophil-deficient mice was found to induce interleukin (IL)-17 production and promote B-cell influx, thereby facilitating tumor progression [56]. LPS activates the NF-κB-IL-6-STAT3 axis to induce inflammation [57], and promotes EMT in biliary epithelial cells via toll-like receptor 4-NF-κB signaling [58,59]. Furthermore, dysbiotic microbiota-derived LPS can drive chemokine-dependent accumulation of monocyte-like macrophages, establishing a precancerous inflammatory niche [60]. Within this inflammation-rich milieu, macrophages may shift from an anti-tumor M1-like phenotype toward a pro-tumor M2-like phenotype, thereby contributing to immune suppression and promoting tumor invasion and angiogenesis [61,62].

#### 2.4.2. Promotion of a Hypoxic Microenvironment

Hypoxia, a hallmark of the TME, is closely associated with tumor metastasis [63,64]. Hypoxia can induce EMT by modulating EMT signaling pathways, activating key transcription factors, and regulating miRNA networks, thereby further enhancing the metastatic and invasive capabilities of tumor cells [65]. The microbiota and its metabolites can exacerbate this process. Persistent colonization of *Helicobacter pylori* in gastric tumor tissues promotes the accumulation of metabolites such as lactic acid, adenosine, and nitric oxide, thereby disrupting pH and oxygen homeostasis in the TME and exacerbating hypoxia-related microenvironmental stress, which may create conditions favorable for tumor progression and metastasis [66]. In addition, tumor-resident *Staphylococcus nepalensis* and *Staphylococcus capitis* can produce lactate. This metabolite upregulates MCT1 expression in tumor cells to facilitate lactate uptake and activate pseudohypoxia signaling, a pathway that has been shown to promote LC metastasis both in vitro and in vivo [67]. Although hypoxia and acidosis are closely linked and frequently coexist in the TME, they are discussed separately for clarity. In many solid tumors, hypoxia promotes glycolytic reprogramming and lactate accumulation, thereby contributing to extracellular acidification [68].

#### 2.4.3. Promotion of an Acidic Microenvironment

Cancer cells preferentially rely on glycolysis for energy production, a phenomenon known as the Warburg effect. This process generates large amounts of lactic acid, contributing to an acidic TME [69]. Acidosis has been strongly associated with increased tumor metastasis and poor clinical prognosis [70,71,72,73]. Owing to the shift toward glycolysis and subsequent lactate accumulation, cancer cells reshape the microenvironment by stimulating angiogenesis through the activation of hypoxia-inducible factor-1α, upregulating vascular endothelial growth factor and its receptors, and exacerbating extracellular acidification. This alteration of the microenvironment in turn may allow cancer cells to evade immune surveillance and further support metastatic dissemination [74]. In addition, cancer cells that have undergone EMT exhibit enhanced glycolysis, characterized by increased glucose uptake and lactate production, which supports their invasive and metastatic features [75]. Notably, the microbiota can accelerate glycolysis. For example, *F. nucleatum* upregulates ENO1 expression via lncRNA ENO1-IT1, which recruits KAT7 histone acetyltransferase, thereby accelerating glycolysis in CRC [76]. Similarly, *Candida albicans* enhances glycolysis in macrophages, as indicated by increased glucose uptake, pyruvate levels, lactate production, and ATP generation [77]. *Acinetobacter baumannii* increases nicotinic acid synthesis to boost nicotinamide adenine dinucleotide metabolism in GC cells. This, in turn, reduces oxidative phosphorylation, increases glycolysis, and activates the hypoxia-inducible factor-1 pathway, ultimately promoting GC metastasis in vitro and in vivo [78]. Together, these findings suggest that microbiota-driven acidification may act in concert with hypoxia to generate a metastasis-permissive TME.

#### 2.4.4. Promotion of an Immunosuppressive Microenvironment

The microbiota can shape an immunosuppressive microenvironment by inducing chronic inflammatory responses and suppressing antitumor immune responses, thereby facilitating tumor progression [79]. For instance, *F. nucleatum* can suppress the activity of natural killer cells and T cells in tumor settings through the interaction between its Fap2 protein and the inhibitory receptor TIGIT, thereby facilitating tumor immune escape [80]. In esophageal squamous cell carcinoma, intratumoral *Lactobacillus* has been associated with increased PD-L1 expression and a higher abundance of tumor-associated macrophages, suggesting a role in strengthening local immunosuppression [81]. In head and neck squamous cell carcinoma, high intratumoral microbiota abundance is associated with an immunosuppressive TME marked by neutrophil enrichment and reduced adaptive immune cell infiltration, and has been linked to immunotherapy resistance in preclinical models [82]. Conversely, in CRC mouse models, elimination of intratumoral microbiota has been shown to convert immune “cold” tumors into “hot” tumors, thereby improving responses to immunotherapy [83].

## 3. Potential Roles of the Microbiota in Tumor Cell Dissemination Through the Circulation

The bloodstream is a major route for tumor cell dissemination, and the microbiota may also contribute to hematogenous metastasis. Oral microbes such as *F. nucleatum* have been detected in multiple tumor types, including CRC and BC. This observation suggests that the hematogenous route is a potential pathway for microbial dissemination [84]. Through several proposed mechanisms, the microbiota may influence tumor cell survival and dissemination in the circulation.

### 3.1. Microbial Signatures in the Circulation

The bloodstream was once thought to be sterile. However, advanced detection technologies have revealed the presence of microorganisms in the bloodstream. A large population study found no evidence of a common blood microbiome in healthy individuals [85]. In contrast, in cancer patients, distinct circulating microbial signatures have been reported and are proposed to contribute to tumor dissemination. However, whether these findings represent a stable blood microbiome remains unclear, and their interpretation requires caution due to the low biomass of blood samples. Furthermore, circulating microbial DNA has emerged as a potential non-invasive biomarker for assessing tumor progression and predicting clinical outcomes [86].

Circulating microbial signals in blood may arise from diverse sources, including translocation of commensals from the oral cavity, skin, or gut; pathogenic infection; release of microbial DNA from necrotic tumor cells; and microbe-containing extracellular vesicles [87,88]. Microbiota from different sites may enter the bloodstream through mechanisms such as vascular barrier disruption, and subsequently influence metastatic progression [89]. Reported blood microbial signatures associated with various solid cancers are summarized in Table 1.

### 3.2. Role of the Microbiota in Tumor Hematogenous Metastasis

**Figure 2:**
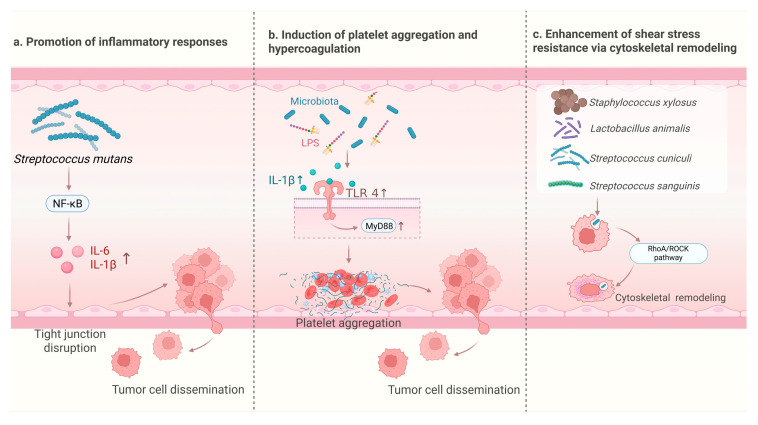
Representative mechanisms by which the microbiota may enhance hematogenous metastasis. The bloodstream serves as a critical route for tumor dissemination, and the microbiota may influence metastatic progression during circulation. Proposed mechanisms include: (1) promotion of inflammatory responses; (2) induction of platelet aggregation and hypercoagulation; and (3) enhancement of shear stress resistance via cytoskeletal remodeling. Created in BioRender. Yuhang, F. (2026) https://BioRender.com/hjoczr6 (accessed on 27 March 2026).

Tumor cell circulation through the bloodstream is influenced by multiple factors, including intensified inflammatory responses, enhanced platelet aggregation, and resistance to fluid shear stress. Preclinical studies indicate that the microbiota may modulate each of these processes, as illustrated in Figure 2.

#### 3.2.1. Promotion of Inflammatory Responses

Elevated levels of circulating inflammatory mediators are associated with poor prognosis in multiple cancers, including ovarian cancer, LC, esophageal cancer, and CRC [107,108,109,110,111]. Cytokines such as IL-6 and IL-8 contribute to tumor progression by upregulating inflammatory responses [112]. Dysbiosis may contribute to systemic inflammation. This leads to elevated circulating levels of inflammatory cytokines, including IL-6 and tumor necrosis factor-α [113]. A representative example is *Streptococcus mutans*. Upon entering the bloodstream, *Streptococcus mutans* can activate the NF-κB signaling pathway and induce the production of pro-inflammatory cytokines such as IL-6 and IL-1β. These changes may impair the tight junctions between vascular endothelial cells, enabling tumor cell extravasation and potentially promoting tumor metastasis [114].

#### 3.2.2. Induction of Platelet Aggregation and Hypercoagulation

Platelets are closely associated with tumor metastasis. In 1968, Gasic and colleagues first showed that thrombocytopenia diminishes tumor metastasis in mice [115]. Platelets promote metastasis by shielding tumor cells from fluid shear stress, facilitating immune evasion, promoting extravasation via ATP release, and recruiting granulocytes for early metastatic niche formation [116,117,118,119]. Bacterial toxins can act as platelet agonists and directly interact with platelets to enhance their activation [120,121]. In addition, LPS can promote platelet aggregation through multiple pathways, including activating the IL-1β autocrine loop, mediating toll-like receptor 4 activation, and triggering the MyD88 signaling pathway [122,123,124]. These effects may contribute to a pathological coagulation state, potentially accelerating metastatic progression [125].

#### 3.2.3. Enhancement of Shear Stress Resistance via Cytoskeletal Remodeling

The intratumoral microbiota has been reported to protect tumor cells from fluid shear stress during hematogenous metastasis, thereby facilitating their survival in the circulation [126]. For example, intracellular microbiota such as *Staphylococcus xylosus*, *Lactobacillus animalis*, *Streptococcus cuniculi*, and *Streptococcus sanguinis* have been linked to modulation of the RhoA/ROCK pathway. This modulatory effect may enable tumor cells to adopt a morphological phenotype that is more resilient to intravascular hemodynamic stress, thereby enhancing their survival by protecting them from mechanical apoptosis [16,127].

## 4. Potential Roles of the Microbiota in Distant Seeding and Colonization

In 1889, Stephen Paget proposed the “seed and soil” hypothesis, positing that certain tumor cells (the “seeds”) metastasize and colonize organs with a suitable growth environment (the “soil”) [128]. In 1980, Ian Hart and Isaiah Fidler further demonstrated that tissue cells can modulate the propensity of malignant tumors to metastasize to specific organs [129]. Notably, metastatic tumors have also been reported to be enriched in microbiota [130]. A 2017 study published in *Science* revealed that the microbiota colonizing primary and metastatic lesions of CRC exhibits high similarity, suggesting an association between microbial colonization and tumor metastasis [131]. These findings raise the possibility that specific microbial activities may shape the microenvironment of distant metastatic sites, thereby potentially facilitating metastatic colonization.

**Figure 3:**
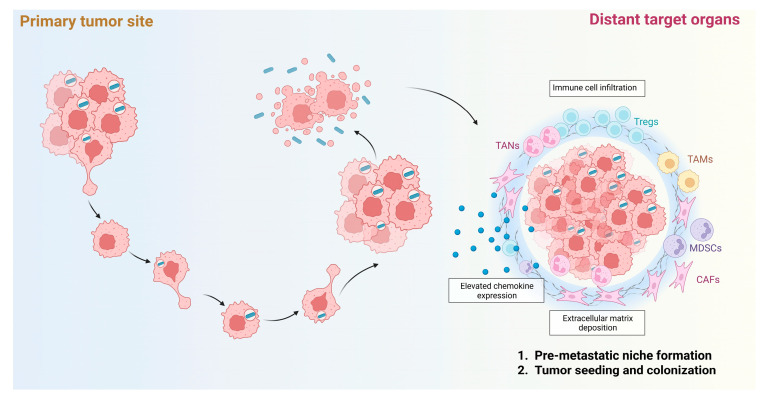
The microbiota may facilitate distant tumor colonization through pre-metastatic niche establishment. In distant organs, the microbiota may remodel the local microenvironment by inducing elevated chemokine expression, immune cell infiltration, and extracellular matrix deposition, thereby creating a permissive niche that serves as a favorable “soil” for tumor cell seeding and colonization. Created in BioRender. Yuhang, F. (2026) https://BioRender.com/hjoczr6 (accessed on 27 March 2026).

The pre-metastatic niche (PMN) refers to a microenvironment characterized by immunosuppression and high vascular permeability, which is induced by the primary tumor in distant organs. Prior to tumor metastasis, the primary tumor may establish the PMN in the target organs, thereby creating a “soil” that is more permissive for tumor colonization and facilitating tumor metastasis [132,133]. Evidence from preclinical models of CRC liver metastasis suggests that specific microbial taxa may contribute to hepatic PMN formation. The presence of microbiota in target organs has been associated with increased cytokine expression and recruitment of myeloid-derived immune cells in target organ tissues [89], both of which are key features of PMN establishment [134,135]. Transcriptomic profiling of target organs harboring microbiota has revealed molecular signatures resembling those of established PMNs, including elevated chemokine expression and extracellular matrix deposition, concurrent with immune cell infiltration. In contrast, in mice treated with antibiotics, diminished microbiota abundance within target organs was accompanied by marked downregulation of tumor necrosis factor-α, transforming growth factor-β, and C-C motif chemokine ligand 2, as well as reduced immune cell infiltration [89]. These findings are summarized in Figure 3.

In addition, preclinical studies have shown that *F. nucleatum* enhances hepatic infiltration of myeloid-derived suppressor cells, reduces the numbers of natural killer cells and T cells, and increases regulatory T-cell abundance, thereby potentially contributing to an immunosuppressive hepatic PMN and promoting CRC metastasis [136]. Similarly, chronic pulmonary infection with *Pseudomonas aeruginosa* or *Staphylococcus aureus* reshapes the lung immune landscape by recruiting tumor-promoting MHCII^hi^ neutrophils, a process that may facilitate PMN formation and contribute to BC lung metastasis [137].

## 5. Limitations and Outstanding Questions

### 5.1. Overview of the Current Evidence Landscape

Overall, current evidence suggests that the microbiota may influence multiple steps of the metastatic cascade, but the strength of support remains uneven. Most findings derive from preclinical models or correlative clinical studies, whereas direct causal evidence in humans is still limited. Although in vitro and animal studies have provided important mechanistic insights, their relevance to human metastatic progression requires further validation. In addition, challenges in causal inference, low-biomass contamination control, and reproducibility warrant cautious interpretation of reported associations. At present, conclusions in this field should therefore be regarded primarily as associative observations or preclinical mechanistic findings, rather than definitive evidence of causation in humans.

The available literature is also unevenly distributed across cancer types and biological settings. Therefore, Table 2 summarizes the core characteristics of the current evidence linking the microbiota to metastasis across key dimensions, including study design, cancer coverage, microbial compartments, and the nature of human evidence.

### 5.2. Limitations of Existing Research

First, much of the evidence from human studies remains correlative rather than causal. Altered microbial abundance and composition are associated with metastatic progression and poor prognosis, but correlation does not equate to causation. Microbial shifts detected in advanced or metastatic patients may represent a trigger, a promoting factor, or simply a secondary change in advanced disease, and this distinction remains unresolved. Furthermore, mechanistic data on microbial regulation come almost exclusively from in vitro and murine models of specific cancer types; their translatability to human metastatic processes is unproven.

Second, low-biomass sample contamination is a pervasive methodological challenge across tumor and blood microbiome research [138]. The extremely low microbial load in tumor and blood samples leaves them susceptible to contamination from reagents, sampling procedures, laboratory environments, and sequencing batches [139,140]. For some cancer types, the very existence of a detectable tissue-resident microbiota remains controversial [141,142]. Additionally, current blood-based assays cannot distinguish viable bacteria from cell-free microbial nucleic acids, so the biological activity and functional relevance of detected signals remain unclear [143]. These factors collectively compromise the reliability and evidential strength of many reported findings.

Third, pro-metastatic microbial effects are not universal. The impact of the microbiota on tumors is highly context-dependent and variable across individuals [144]. The same bacterial species can exert divergent effects across different hosts, tumor types, or treatment stages. Current evidence thus supports a nuanced view: it is not the microbiota as a whole that universally drives metastasis, but specific taxa, strains, metabolites, or microbe-induced host responses that modulate metastatic processes under particular conditions. Findings should therefore be interpreted in light of study design, sample type, and validation rigor, and not overgeneralized.

Fourth, microbe-mediated metastasis does not follow a single unified mode of action. Diverse microorganisms act on tumor cells and the TME via distinct effector molecules, including secreted metabolites, virulence factors, and cell-associated structural components [145]. Despite these divergent upstream mechanisms, downstream effects often converge on shared pro-metastatic programs: inflammatory activation, immune modulation, EMT induction, TME remodeling, and PMN formation, among others. The interplay between species/strain-specific mechanisms and these common pathways remains poorly characterized, and mechanistic data remain fragmented. Future research should disentangle taxon-specific mechanisms from core shared pathways to support precise microbiome-targeted anti-metastatic strategies.

### 5.3. Unresolved Scientific Questions

Several key scientific questions remain open. First, it remains unclear whether specific microbial alterations are a cause, co-promoter, or consequence of cancer progression. Second, the origin of microbial signals detected in tumors and blood requires clarification, including whether they derive from viable bacteria, host cell-associated microbial components, or residual DNA from bacterial lysis. Third, how species- or strain-specific mechanisms intersect with shared pro-metastatic pathways remains poorly understood.

Answering these will require studies with long-term follow-up, strict contamination control, standardized analytical pipelines, and functional validation, to reliably separate correlative observations from causal mechanisms.

## 6. Conclusions and Perspectives

**Figure 4:**
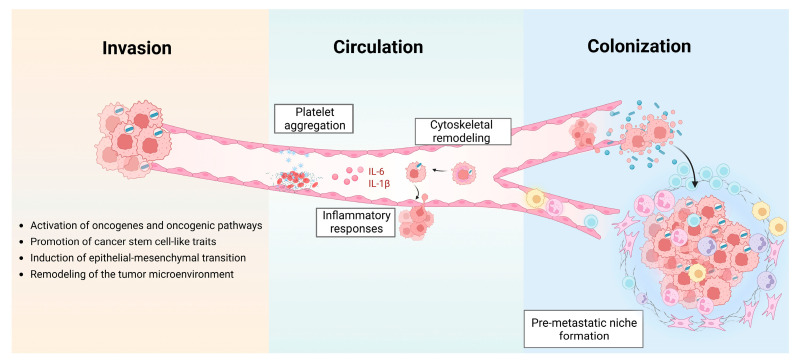
Potential roles of the microbiota across the tumor metastatic cascade. Schematic illustration of how the microbiota may participate in different stages of tumor metastasis, including invasion, circulation, and colonization. During invasion, the microbiota may enhance cancer cell invasiveness by activating oncogenes and oncogenic pathways, promoting cancer stem cell-like traits, inducing epithelial–mesenchymal transition, and remodeling the tumor microenvironment. During circulation, the microbiota may influence metastatic progression by promoting inflammatory responses, inducing platelet aggregation and hypercoagulation, and enhancing resistance to fluid shear stress via cytoskeletal remodeling. During colonization, the microbiota may contribute to pre-metastatic niche formation and thereby support metastatic seeding and outgrowth in distant organs. Created in BioRender. Yuhang, F. (2026) https://BioRender.com/hjoczr6 (accessed on 27 March 2026).

In summary, this review synthesizes current evidence linking the microbiota to multiple steps of the metastatic cascade in specific biological contexts, including local invasion, hematogenous dissemination, and distant colonization (Figure 4). Notably, most mechanistic insights derive from preclinical models.

On balance, the microbiota is best framed as an emerging, context-dependent modifier of metastasis, rather than a universal driver. This framing reflects the current evidence base, which is dominated by clinical associations and preclinical mechanistic studies, whereas direct causal evidence in humans remains limited. Its effects are shaped by multiple factors: tumor type, host immune status, anatomical location, and specific microbial taxa or strains. It should therefore not be described as having a universal pro-metastatic effect; instead, its role must be interpreted within specific biological contexts.

Importantly, while this review focuses on the pro-metastatic potential of the microbiota, host-microbe interactions are not uniformly tumor-promoting. Under specific conditions, certain commensal communities may suppress tumorigenesis or slow progression through immune modulation, metabolic homeostasis maintenance, and anti-inflammatory effects [146]. Future work should avoid a simplistic “pro-tumor/anti-tumor” dichotomy, and instead characterize microbial roles in a nuanced, dynamic manner, grounded in specific tumor types, host states, and microecological backgrounds.

With rigorous contamination control and standardized sampling and analytical workflows, future research integrating longitudinal cohorts, functional experiments, and appropriately designed interventional studies will help clarify whether microbial factors act as drivers, co-promoters, or bystanders of disease progression. Such work will not only deepen our understanding of metastatic biology, but also advance the discovery of microbe-associated metastatic biomarkers and the development of microbiome-targeted anti-metastatic strategies.

## Figures and Tables

**Table 1:** Reported Circulating Microbial Signatures in Solid Tumors.

Cancer Type	Microorganism(s)	Change	Clinical Association	References
Colorectal Cancer	Microbial diversity	Inconsistent findings	No consistent conclusion regarding changes compared to healthy individuals Responders to immunotherapy may exhibit higher diversity	[90,91,92]
	*Fusobacterium nucleatum*	Increased	Enriched in postoperative samples	[93]
	*Butyricimonas*, *Parabacteroides*, *Odoribacter*, *Shigella*, *Hungatella*	-	Demonstrates diagnostic potential compared to other cancer types	[94]
Pancreatic Cancer	*Verrucomicrobia*, *Deferribacteres*, *Bacteroidetes* (Phylum level)	Increased	-	[95]
	*Actinobacteria* (Phylum level)	Decreased	-	
	*Ruminococcaceae UCG-014*, *Lachnospiraceae NK4A136 group*, *Akkermansia*, *Turicibacter*, *Ruminiclostridium*, *Lachnospiraceae UCG-001* (Genus level)	Increased	-	
	*Stenotrophomonas*, *Sphingomonas*, *Propionibacterium*, *Corynebacterium* (Genus level)	Decreased	-	
Ovarian Cancer	*Acinetobacter* (Genus level)	Increased	Potential tool for ovarian cancer diagnosis	[96]
Gastric Cancer	*Acinetobacter*, *Bacteroides*, *Haemophilus parainfluenzae*	Predominant taxa	Potential tool for gastric cancer diagnosis	[97]
Hepatocellular Carcinoma	Microbial diversity	Decreased	Compared to cirrhosis and healthy individuals	[98]
	*Proteobacteria* (Phylum level)	Increased	-	
	*Staphylococcus*, *Acinetobacter*, *Klebsiella*, *Trabulsiella* (Genus level)	Increased	-	
Breast Cancer	Microbial diversity	Increased	Compared to healthy individuals	[99]
	*Gammaproteobacteria*, *Clostridia*, *Bacteroidia*, *Negativicutes*, *Coriobacteriia* (Class level)	Increased	-	[100]
	*Bacilli*, *Actinobacteria*, *Alphaproteobacteria*, *Oxyphotobacteria* (Class level)	Decreased	-	
	*Bacillus* (Genus level)	Increased (4-fold)	In patients concurrently diagnosed with thyroid tumors compared to those with normal thyroid	[101]
	*Kluyvera* (Genus level)	Increased (8.1-fold)		
Melanoma	Microbial diversity	Decreased	Compared to healthy individuals	[102]
	*Castellaniella* (Genus level)	Decreased	Detected only in the healthy control group	
Lung Cancer	*Candidatus_Babela*, *Methanotorris*, *Anaeromusa*, *Hirschia*, *Parascardovia*, *Agreia*	-	High abundance associated with poor prognosis	[103]
	*Kozakia*, *Andromedalikevirus*, *Natronococcus*, *Demequina*, *Desulfuromonas*, *Blastococcus*, *Anaerobacillus*, *Ewingella*	-	High abundance associated with favorable prognosis	
	*Peptostreptococcae*, *Paludibaculum*, *Lewinella*	-	Correlated with response and clinical benefit	[104]
	*Gemmatimonadaceae*	-	Correlated with tumor progression	
Esophageal Cancer	*Actinobacteria*, *Bacteriodetes*, *Firmicutes*, *Proteobacteria* (Phylum level)	Predominant phyla	-	[105]
	*Bifidobacterium*, *Escherichia*, *Jerseyvirus*, *Prevotella* (Genus level)	Predominant genera	-	
Brain Tumor	*Firmicutes*, *Proteobacteria*, *Bacteroidota* (Phylum level)	Predominant phyla	Preliminary observation	[106]
	*Enterococcus*, *Klebsiella*, *Blautia*, *Lactobacillus*, *Clostridium innocum* group, *Ruminococcus* (Genus level)	Predominant genera		

**Table 2:** Main characteristics and limitations of the current evidence linking the microbiota to tumor metastasis.

Evidence Dimension	General Characteristics of the Current Literature	Main Limitations and Considerations
Study design	The current literature is dominated by pre-clinical mechanistic studies, supplemented by a limited number of clinical association analyses.	Direct causal evidence in humans remains limited.
Cancer coverage	Relevant evidence has been reported across multiple solid tumors, including colorectal, breast, gastric, lung, and esophageal cancers, with colorectal cancer representing a relatively more extensively studied setting.	The strength and depth of evidence vary across cancer types and remain unevenly distributed.
Microbial compartments	Reported findings involve gut-associated, oral-associated, tumor-associated, and blood-derived microbiota, but the strength of evidence linking these different microbial compartments to metastasis is not equivalent.	Tumor and blood samples are typically low-biomass specimens and are highly susceptible to contamination from reagents, sampling procedures, laboratory environments, and sequencing batches, which may affect result reliability and evidential strength.
Human evidence	Human studies are mainly based on correlative, retrospective, or cross-sectional observations.	Longitudinal investigations and interventional validation remain relatively limited.

## Data Availability

No new data were created or analyzed in this study. Data sharing is not applicable to this article.

## Abbreviations

The following abbreviations are used in this manuscript:

ATP	Adenosine triphosphate
BC	Breast cancer
CRC	Colorectal cancer
CSC	Cancer stem cell
EMT	Epithelial–mesenchymal transition
*F. nucleatum*	*Fusobacterium nucleatum*
GC	Gastric cancer
IL	Interleukin
LC	Lung cancer
LPS	Lipopolysaccharide
NF-κB	Nuclear factor kappa-B
PMN	Pre-metastatic niche
TME	Tumor microenvironment
